# Early Deep Venous Thrombosis After Hip Fracture Surgery in Patients in Pharmacological Prophylaxis

**DOI:** 10.3390/jcm14030726

**Published:** 2025-01-23

**Authors:** Carlo Rostagno, Massimo Gatti, Alessandro Cartei, Roberto Civinini

**Affiliations:** 1 Dipartimento Medicina Sperimentale e Clinica, Università di Firenze, 50134 Firenze, Italy; 2 Cardiologia Generale AOU Careggi, 50134 Firenze, Italy; gattim@aou-careggi.toscana.it; 3 Ortogeriatria AOU Careggi, 50134 Firenze, Italy; carteia@aou-careggi.toscana.it; 4 Ortopedia e Traumatologia Generale AOU Careggi, 50134 Firenze, Italy; roberto.civinini@unifi.it

**Keywords:** venous thromboembolism, low molecular heparin, hip fracture, elderly

## Abstract

**Background:** Venous thromboembolism frequently complicates orthopedic surgery. The aim of the study was to evaluate the overall incidence, site, and risk factors for venous thromboembolism in patients undergoing hip fracture surgery in DVT prophylaxis according to guidelines recommendations. **Methods:** Standard ultrasonography (CUS) was performed in the 5–6th postoperative day in all patients who underwent hip fracture surgery between 1 January and 31 December 2019. Pharmacological prophylaxis was started within 12 h from admission. In the first half of the year, dalteparin (5000 IU/day) was available while nadroparin (38 IU/kg until 3rd postoperative day and thereafter 57 IU/kg) was available in the second part of the year. **Results:** A total of 505 patients, 144 males and 361 females, with a mean age of 84 years, entered in the study. Post-operative DVT was found at screening ultrasonography in 121 patients (24%). Most involved distal veins (91) while proximal DVT occurred in 30. Two patients had not fatal pulmonary embolism (0.3%). Time to surgery (*p* = 0.0009) and ≥2 comorbidities (*p* = 0.0198) were independent predictive factors of DVT. Moreover, dalteparin prophylaxis was associated with a 1.7-times higher risk of developing a DVT compared to nadroparin. **Conclusions:** DVT occurs in 24% of patients after hip fracture surgery despite thromboprophylaxis. Time to surgery and ≥2 comorbidities were independent risk factors. The protective effects of nadroparin should be confirmed by a randomized trial. All patients with DVT were discharged with indication to anticoagulation for at least three months.

## 1. Introduction

Pharmacologic prophylaxis in patients undergoing surgery for lower limb fractures significantly decreased the incidence of venous thromboembolic disease [1]. Without thromboprophylaxis, although, in most cases, asymptomatic, DVT was reported in nearly 80% of patients with hip surgery [2]. Guidelines suggest as class I, level A recommendation the administration of antithrombotic drugs in patients who need surgery for hip fracture [3]. However, symptomatic deep venous thrombosis (DVT), despite the widespread diffusion of prophylactic measures [4,5,6], still occurs in 1.5–10% of patients undergoing major orthopedic surgery. Fatal pulmonary embolism is relatively uncommon (0.5 to 1.5%) [7,8]. A large review reported an overall incidence of symptomatic VTE of 0.53% for hip surgery; DVT, 0.26%; and pulmonary embolism (PE), 0.14% [9]. Most of the patients developed PE or DVT within the first 72 postoperative hours; nevertheless, data from the FAITH and HEALTH trials suggested that about 40% were diagnosed more than 6 weeks after surgery [10]. The authors suggested that treatment with arthroplasty rather than fixation was associated with increased incidence of VTE. Bone cement implantation syndrome may account for a small number of early pulmonary embolism cases, not rarely fatal, that should be differentiated from “thrombotic venous embolism” [11]. The American College of Chest Physician Guidelines does not suggest routine ultrasonography after surgery; therefore, only a few studies have systematically evaluated the incidence of DVT after hip fracture surgery before discharge in patients undergoing DVT prophylaxis. The aim of the present investigation was to evaluate the overall incidence, site, and risk factors for DVT in consecutive patients referred for the surgical treatment of hip fracture who underwent pharmacological prophylaxis according to guidelines recommendations.

## 2. Materials and Methods

### 2.1. Patient Population

The study is part of a project of the Italian Health Ministry and Regione Toscana—RF-2010-2316600—and was approved by the Ethical Committee of Regione Toscana. Patients referred to the Trauma Center of the Azienda Ospedaliera Universitaria Careggi Firenze between 1 January and 31 December 2019 for surgical treatment of hip fragility fracture were included in the study. The only exclusion criterion was the conservative treatment of the fracture. Informed consent to treatment and collection of clinical data for research purposes was given at hospital admission.

### 2.2. VTE Prophylaxis

Pharmacological prophylaxis of venous thromboembolism was started with low molecular weight heparin within 12 h of admission according to ACCP guidelines [3]. In the first half of the year (1 January–31 July), dalteparin (administered at a single s.c. dose of 5000 IU/day) was available for VTE prophylaxis while nadroparin (administered at a single s.c. dose of 38 IU/kg until 3rd postoperative day and thereafter at the dose of 57 IU/kg) was available in the second part of the year (1 August–31 December). These groups of similar numerosity and overlapping clinical and demographic characteristics allowed to compare the relative effectiveness of the two molecules.

### 2.3. Ultrasonographic Examination

Compression ultrasonography standard B-mode method (CUS) was performed in the 5–6th postoperative day. Portable Doppler ultrasonography (Alpha, Esaote, Firenze, Italy) with high-resolution linear probes 5–7.5 MHz was used for examination. Ultra-sonographic study included superficial and deep femoral veins, popliteal veins, and calf veins (posterior tibial, peroneal, gastrocnemius, and soleal). The main diagnostic criterion for proximal or distal DVT was the absence of compressibility in the transverse plane by CUS associated with the absence of color Doppler flow after distal muscle compression. Diagnosis of DVT was made when solid echoes were noted within veins or when the venous lumen was not completely or only partially compressed by the CUS method.

### 2.4. Data Collection

The type of fracture according to AO/Orthopedic Trauma Association (AO/OTA) classification, time to surgery, clinical status at hospital admission, demographic data, presence of comorbidities, postoperative anemia, need for transfusion, and drug used for thrombosis prophylaxis were recorded and analyzed to evaluate the relation with the development of DVT.

### 2.5. Statistical Analysis

Values for continuous variables were given as the mean plus/minus standard deviation (SD) and compared by Student’s *t* test. Categorical variables are reported as frequency and percentage. Categorical variables were compared using the chi square test or the Fisher exact test. After the univariate analyses, a logistic multivariable regression analysis was performed to identify independent predictors for development of DVT. Because of multiple testing, only variables with a two-sided *p* < 0.05 in the univariate analysis were accepted for the model. Statistical analysis was performed with the use of a statistical software program (SPSS 26, Inc., Chicago, IL, USA). A probability value of <0.05 was statistically significant.

## 3. Results

The study entered 505 patients, 144 males (28%) and 361 females (72%), with a mean age of 84 years (range 70–103). A total of 135 of the patients (27%) had <4 ADL preserved. A history of dementia was found in 83 patients and 48 had Parkinson’s disease. The clinical characteristics are reported in Table 1.

Fractures were classified according to OTA classification. A total of 246 (48%) were femoral neck fractures (B1B2 OTA), 235 (46%) were trochanteric fractures (A1, A2 OTA), and 24 (6%) were intertrochanteric fractures (A3 OTA). Osteosynthesis with canulated screws was performed in 42 cases, osteosynthesis with slipping screw-plate in 50 cases, and osteosynthesis with a medullar nail in 230 cases; a cephalic prosthesis was implanted in 168 patients and 15 underwent total hip replacement. Time to surgery was on average 3.3 days (range 0–12). A total of 55% of the patients underwent surgery < 48 h after the fracture. Post-operative anemia < 9 g/dL was found in 220 cases (43%) and 190 patients needed RBC transfusions (37%).

Low-molecular-weight heparins (LMWH) were used for DVT thromboprophylaxis in 498 patients (nadroparin in 184 patients and dalteparin in 266 patients). Among the 17 remaining, 10 patients received fondaparinux (in most cases, for LMWH-induced thrombocytopenia or previous adverse reactions to LMWH) and seven patients were given dose-adjusted warfarin sodium by standardized prescriptive protocol according to the results of laboratory monitoring of the prothrombin time and according to a predefined warfarin nomogram.

A post-operative DVT was found in 121 patients (24%). It was localized in the calf vessels in 65 patients, in the sub-popliteal vessels in 26, in the popliteal vessels in 15, and finally, in the proximal vessels in 15. In 27 patients, the DVT was contralateral to the fracture and in 24 cases, it was bilateral. During the hospital stay, two non-fatal pulmonary embolisms occurred (0.3%). The hospital stay was on average 13.3 days. Patients with DVT were older (86.8 vs. 82.7 years, *p* = 0.0038), while we did not find any significant correlation between DVT and gender or fracture type. Time to surgery was significantly longer in patients who developed DVT (5.2 days vs. 2.9 days). No significant relation was found between the type of surgery and DVT. The analysis of the DVT risk factors showed a significant association with age (*p* < 0.03), time to surgery (*p* < 0.001), compromised BADL (<4) (*p* < 0.0001), dementia (*p* < 0.0001), chronic heart failure (*p* < 0.0001), atrial fibrillation (*p* < 0.01), COPD (*p* < 0.0006), transfusions (*p* < 0.04), and the coexistence of two or more major comorbidities (*p* < 0.0001) (Table 2).

**Figure 1:**
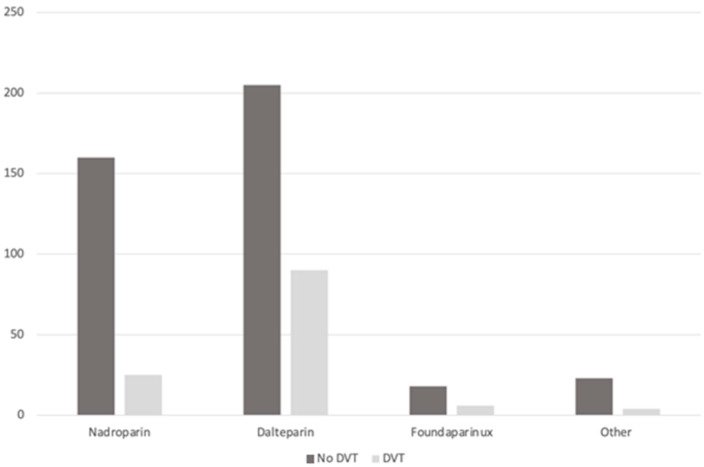
Incidence of DVT in relation to pharmacological prophylaxis.

The two groups treated with different low molecular weight heparin did not differ with age, gender, type of fracture, and surgery. No significant differences were found, also, for comorbidities. DVT was more frequent in the dalteparin group than in the nadroparin group (27% vs. 9.8% patients, *p* < 0.0001) (Figure 1). Logistic multivariate regression analysis showed that time to surgery (*p* = 0.0009) and the presence of two or more comorbidities (*p* = 0.0198) were independent predictive factors for the risk of DVT. Moreover, dalteparin prophylaxis was associated with a 1.7-times higher risk of developing DVT compared to nadroparin prophylaxis (Table 3).

## 4. Discussion

Current guidelines suggest against routine Doppler examination in patients with trauma; moreover, still debated is the type and duration of pharmacological prophylaxis [12]. However, late occurrence of pulmonary embolism, not rarely fatal, has been reported after trauma [13] and this was more frequent in patients with mobility-limiting lower extremity injuries with an Injury Severity Score < 15. Most of these patients were asymptomatic and did not undergo adequate prophylaxis [14].

The incidence of DVT diagnosed by ultrasound after hip fracture surgery has been reported between 20 and 42%. Proximal DVT ranged from 4 to 7.2% [15]. Significant difference was related to the site of fracture, with higher risk for femoral neck fractures [16]. It must be underlined that ultrasound examination found signs of DVT in a not negligible number of patients before surgery [17,18]. Wang et al. [18] reported an overall incidence of postoperative DVT in 48% of patients after lower limb trauma. About 30% of cases were present before surgery. Distal DVT accounted for 88% of overall diagnosed DVT cases. DVT was more frequent in patients with fracture above the knee, with most suffering from hip fracture.

Preoperative DVT was found in 20% of patients (96% distal). Again, femur fractures had a higher incidence, and delay of surgery > 48 h increased the risk of DVT by five folds. No mention to prophylaxis is reported. Another study from China tested several risk factors for DVT after hip fracture surgery: Only six demonstrated an independent effect on DVT occurrence, including history of a VTE event, time from injury to DVT screening, BMI, peripheral vascular disease, reduced albumin, and D-Dimer > 1.0 mg/L [19]. Similar results were reported by a recent meta-analysis. Female sex and medical comorbidities were also associated with a higher risk [5]. Few data exist about the type of fracture and, therefore, the intervention performed and the risk of VTE. Data from the FRAILT and HEALTH studies suggested that arthroplasty was associated with a more than two-fold increased incidence of VTE in comparison to fixation, in particular, in subjects who underwent total hip arthroplasty [10]. This was not confirmed by other studies [19].

Our study found that age, time to surgery, compromised functional condition expressed by more than two compromised BADL, chronic heart failure, atrial fibrillation, COPD, the need for transfusions, and finally, the coexistence of two or more major comorbidities were related with a higher risk of DVT. However, only time to surgery and the presence of two or more comorbidities were independently associated with development of DVT. We were not able to find any significant difference in DVT incidence with type of fracture and related surgery. The increased risk of VTE related to multiple medical comorbidities confirmed the results of previous investigations [20,21].

Although calf DVT is usually considered a low-risk condition, including in a study by Chen et al. [22], multivariate Cox analysis showed that calf DVT was significantly associated with a three-fold increased risk of 30-day mortality in comparison to patients without DVT.

The results from the present study show that the incidence of deep vein thrombosis after hip fracture surgery is above 20%, and in one third of cases, involved proximal vessels, a number significantly lower in comparison to previous investigations [13,16,17]. The incidence of pulmonary embolism was 0.3%. Time to surgery was the main risk factor for the development of DVT despite the early introduction of prophylaxis, within 12 h from trauma, outlining that early surgery is associated with an overall better outcome in patients undergoing surgery for hip fracture [23,24]. The finding of a higher risk of DVT related to surgical delay agrees with a recent observation by Taoka et al. [25]. Among 862 patients underwent hip fracture surgery in a 10-year period, 484 were found to have DVT (436 distal and 46 proximal). Delay to surgery > 48 h and age were the only independent factors related to DVT at multivariate analysis. They did not find any relation with the anticoagulant drug employed for prophylaxis.

The literature data suggest that at least half of DVT cases in patients with lower limb fractures may be detected before surgery, but almost all studies were performed in the absence of prophylactic measures. We start thromboprophylaxis at hospital admission and preoperative ultrasonography has been reserved for patients with symptoms. Therefore, the incidence of preoperative DVT in this group of treated patients is unknown. A long delay to intervention favors both preoperative and postoperative DVT in fragility fractures. Early starting of thromboprophylaxis should decrease the overall incidence of symptomatic VTE, preventing thrombus formation during the whole perioperative period. The overall lower incidence of DVT found in the present study supports the role of thromboprophylaxis after fracture.

A combination of mechanical compression and low molecular weight heparin, started as soon as possible after trauma, if non-contraindicated, is the more frequent strategy for the pharmacological prophylaxis of VTE in patients with hip fracture. DOAC or fondaparinux, started after surgery, aspirin, or non-pharmacological methods are alternative methods suggested by guidelines [3]. Recently, aspirin showed a renewing interest, due to lower bleeding risk compared to anticoagulants. A recent meta-analysis reported a similar effect on overall mortality and the prevention of VTE in comparison with other agents following surgical intervention for hip fractures [26]. However, the authors consider this less than robust due to most of the studies included being less statistically robust, with more than half of the studied outcomes considered statistically fragile. Moreover, the lower bleeding risk has also been questioned [27,28].

There are no studies comparing the effects of the various low molecular weight heparin in DVT prophylaxis after hip fracture surgery. Different enoxaparin dosages have been compared in a meta-analysis including 44 randomized studies in 56,423 patients. A total of 35 were double-blind investigations (54,117 patients). In pooled studies including more than 50,000 patients, enoxaparin 3000 anti-Xa IU twice daily was associated with a reduced risk of venous thromboembolism in comparison to enoxaparin 4000 anti-Xa IU once daily (relative risk [RR]: 0.53, 95% confidence interval [CI]: 0.40 to 0.69), but was associated with an increased risk of major bleeding [29]. The introduction of heparin as a substitution for aspirin significantly reduced the rate of symptomatic DVT (1.62% vs. 0.83%, *p* < 0.05) [30]. The present study was not aimed at comparing the effects of different heparin regimens; however, the change in July of the molecule supplied to hospital allowed to obtain two different groups matched for age, gender, the type of fracture, and comorbidities. Dalteparin and nadroparin showed significantly different protective efficacies against the occurrence of DVT, with a total prevalence of 27% and 9.8%, respectively. The difference between the two molecules can be related to the different dose regimens; dalteparin was used with a fixed dose while the Nadroparin dose was increased on the third post-surgery day.

The study was designed to evaluate the incidence of early post-operative DVT after hip fracture surgery in patients undergoing standard thromboprophylaxis. We do not recommend routine predischarge ultrasound examination, the usefulness of which has been questioned after the demonstration by a randomized prospective study of the absence of clinical benefit. However, the observation that even distal DVT was associated with an increased 3-month mortality and a not-negligible risk of pulmonary embolism [22] suggested, in this cohort, the need for prolonged full anticoagulation when DVT was demonstrated before discharge. The results from the study by Utter et al. [31] agree with these suggestions. After hip fracture surgery, 243 patients with calf DVT were treated with anticoagulants while 141, not treated, were considered as control group. Evolution to proximal vessels occurred in seven controls (5.0%) and four anticoagulation recipients (1.6%), while pulmonary embolism occurred in six controls (4.3%) and four anticoagulation recipients (1.6%). Therapeutic anticoagulation was associated with a decreased risk for proximal DVT or PE at 180 days (odds ratio [OR], 0.34; 95% CI, 0.14–0.83); however, this was associated with an increased risk for bleeding.

The difference in the evaluation of legal implications between Europe and United States, with the risk for pulmonary embolism and related mortality weighted more than the more frequent risk of bleeding in Europe, suggests that new large prospective studies are warranted to answer the question of cost-effectiveness of prolonged anticoagulation in patients with post-operative DVT.

Similarly, the observation by chance of a different protective effect of nadroparin in comparison to dalteparin may not be translated in clinical practice and, again, the question of whether there exists a true difference among different LMWHs approved for DVT prophylaxis needs the confirmation of randomized studies.

## 5. Conclusions

More than 20% of the patients surgically treated for proximal femoral fractures develop a DVT detected by screening ultrasonography before the hospital discharge despite LMWH prophylaxis. The delay from trauma to surgery and the presence of comorbidity increases the DVT risk. The different protective effectiveness of nadroparin in comparison to dalteparin may be related to the different overall LMWH dose administered according to the nadroparin therapeutic schedule, which includes an increase in dose at the third post-operative day; moreover, hidden confounders may have influenced our results. Randomized studies will be necessary to compare different LMWHs. The long-term outcomes related to early DVT and the need for prolonged anticoagulation also need further investigations, since available studies offer a clear demonstration that distal DVT may also be associated with the late occurrence of pulmonary embolism.

## Figures and Tables

**Table 1:** Baseline characteristics of patients included in the study.

Age (ys)	84.0 ± 8.0
Timing to surgery < 48 h	264/203
BADL ≤ 4 BADL > 4	137 330
Dementia	17%
Parkinson’s disease	5.4%
Major comorbidities ≥ 2	42%
Cerebrovascular diseases	7%
Coronary artery diseases	37%
Clinical instability	14%
Chronic heart failure	23%
Atrial fibrillation	13%
Cancer	14%
Diabetes	17%
Chronic kidney disease	2.9%

BADL—basic activity of daily living.

**Table 2:** Comparison between clinical characteristics of patients with and without DVT at discharge.

Baseline Characteristics	No DVT (384)	DVT (121)	*p*
M/F	104/280	40/81	0.28
Age (ys)	82.7 ± 10	86.8 ± 9	0.0398
Time to surgery (days)	2.9 ± 2.2	5.2 ± 8.9	<0.001
Fracture type			
B1, B2, B3	198	48	0.0786
A1, A2	169	66	
A3	17	7	
Time to surgery < 48 h	220	52	0.4807
BADL ≤ 4	104	29	<0.0001
BADL > 4	279	89	
Dementia	69	14	<0.0001
Major comorbidities > 2	133	46	<0.0001
Cerebrovascular diseases	65	19	1.0000
Coronary artery diseases	129	47	0.2085
Clinical instability	90	22	<0.0001
Chronic heart failure	43	14	0.0117
Atrial fibrillation	59	19	0.1350
Cancer	71	14	0.0981
COPD	77	10	0.0006
Anemia (Hb < 9 g/dL)	217	68	0.83

**Table 3:** Multivariate logistic regression analysis.

Variable	Odds Ratio	95% CI	*p*
Time to surgery	1.142	1.056–1.23	0.0009
Comorbidities (number)	1.402	1.055–1.863	0.0198
Dalteparin vs. nadroparin	1.729	1.238–2.41	0.0013
Age	0.999	0.974–1.024	0.0925
Clinical instability	1.275	0.604–2.693	0.5231
BADL	0.950	0.782–1.15	0.61

## Data Availability

Data are available on request.
